# Mitochondrial dependent pathway is involved in the protective effects of carboxymethylated chitosan on nitric oxide-induced apoptosis in chondrocytes

**DOI:** 10.1186/s12906-019-2808-x

**Published:** 2020-01-29

**Authors:** Bin He, Fei Wu, Xiaohai Li, Yang Liu, Li Fan, Haohuan Li

**Affiliations:** Department of Orthopaedics, Renmin Hospital of Wuhan University, 238# Jiefang Road, Wuchang District, Wuhan, Hubei 430060 People’s Republic of China

**Keywords:** Carboxymethylated chitosan, Osteoarthritis, Chondrocytes, Apoptosis

## Abstract

**Background:**

Chondrocyte apoptosis activated by the mitochondrial dependent pathway serves a crucial role in cartilage degeneration of osteoarthritis (OA). In the present study, the protective effects of CMCS against sodium nitroprusside (SNP)-induced chondrocyte apoptosis were evaluated and the underlying molecular mechanisms were elucidated.

**Methods:**

Chondrocytes were isolated from articular cartilage of SD rats and identified by type II collagen immunohistochemistry. The chondrocytes stimulated with or without SNP to induce apoptosis, were treated by CMCS for various concentrations. The cell viability were determined by MTT and LDH assays. Cell apoptotic ratio was determined by Annexin V-FITC/PI staining. Mitochondrial membrane potential (ΔΨm) was detected by using Rhodamine123 (Rho123) staining. To understand the mechanism, the mRNA expression levels of Bcl-2, Bax, cytochrome *c* (Cyt *c*) and cleaved caspase-3 were detected by real-time PCR and western blot analysis, respectively.

**Results:**

It was shown using the MTT and LDH assays that CMCS protected the viability of chondrocyte against SNP damage. Annexin V-FITC/PI and Rho123 staining showed that CMCS not only inhibited the cell apoptosis but also restored the reduction of the ΔΨm in chondrocytes. In SNP-induced chondrocytes, CMCS down-regulated the expression of Bax, Cyt *c* and cleaved caspase-3 but upregulated the expression of Bcl-2, as shown by real-time PCR and western blot.

**Conclusions:**

Taken together, these results indicated that CMCS has the protective effect on chondrocytes against SNP-induced apoptosis, at least partly, via inhibiting the mitochondrial dependent apoptotic pathway. Thus, CMCS may be potentially used as a biological agent for prevention and treatment of OA.

## Background

Osteoarthritis (OA) is characterized by degeneration of the articular cartilage and is a major cause of joint dysfunction in the elderly population, is one of the most common chronic disease, affecting an estimated 10% of man and 18% of women over the age of 60 years [1]. Numerous studies have suggested that the main cause of OA is an excessive apoptosis related loss of chondrocytes and degeneration of the cartilage. Although the exact pathogenesis of OA remains poorly understood, there is no doubt that the apoptosis of chondrocyte is the key pathogenic events [2]. Several inflammatory cytokines have been widely investigated to induce chondrocyte apoptosis, such as nitric oxide (NO) [3], Interleukin-1beta (IL-1β) [4], hydrogen peroxide [5] and tumor necrosis factor-α (TNF-α) [6]. Although there is growing interest in medical management of OA, this requires new therapeutic strategies and approaches to deal with OA in this rapidly aging society [2, 7]. Apoptosis was regulated by extrinsic pathway (surface receptor dependent) and intrinsic pathway (mitochondria dependent). One of the important elements involves in these pathways is the activation of caspase-3 by cytochrome *c* (Cyt *c*) that is induced in mitochondria signaling pathway. Based on the important roles of mitochondria-dependent pathway in progress of chondrocyte apoptosis in cartilage degeneration and occurrence of OA [8], inhibition of chondrocyte apoptosis through regulating Bcl-2 family mediated mitochondrial dependent apoptotic pathway has a major therapeutic importance in treatment of articular cartilage degeneration in OA.

Carboxymethylated chitosan (CMCS), the soluble derivative of chitosan, which has many desirable biological and pharmacological properties [9]. In our previous study, we have found CMCS could promote proliferation and inhibit apoptosis in cultured Schwann cells [10–13], we also found CMCS could inhibit the apoptosis of cultured nucleus pulposus cells [14]. CMCS has been widely studied in OA related diseases in recent years in vivo and in vitro in recent days [15–17]. We have also used CMCS in cultured chondrocytes and found CMCS also has the inhibitory effect on NO-induced apoptosis in cultured chondrocytes [18, 19].

Since OA is characterized by degeneration of cartilage, better understanding of the biological effects of CMCS on cartilage would facilitate the development of clinically available therapeutic agents for the treatment of joint diseases. The aim of this study was to clarify whether CMCS is effective in preventing SNP-induced apoptosis and to discuss the potential and advantages of this approach as a therapeutic method for the management of OA.

## Methods

### Animals and reagents

Three weeks old Sprague-Dawley (SD) rats were provided by Experimental Animal Center of Wuhan University Medical School. CMCS (Cat# 83512–85-0) was purchased from Santa Cruz Biotechnology, Inc. (Santa Cruz, CA, USA). Dulbecco’s modified Eagle’s medium (DMEM**;** Cat# 11965092) and fetal bovine serum (FBS; Cat# 26140079) were purchased from Gibco company (USA). Primers were obtained from Invitrogen Biotech Company (USA). MTT (Cat# M2128), Sodium nitroprusside (Cat# BP453) and Rhodamine123 (Cat# 83702) were purchased from Sigma-Aldrich (USA). Lactate dehydrogenase (LDH) assay kit (Cat# C0016) was provided by Beyotime Biotechnology (China). The Annexin V-FITC apoptosis detection kit (Cat# ALX-850-020-KI02) was provided by Enzo Life Sciences (USA). Anti-Bcl-2 (Cat# 2870), anti-Bax (Cat# 5023), anti-Cyt *c* (Cat# 11940) and anti-cleaved caspase-3 (Cat# 9579) antibodies were purchased from Cell Signaling Technology, Inc. (USA). horseradish peroxidase (HRP) conjugated mouse anti-rabbit IgG (Cat# sc-2357), anti-collagen type-2 (Cat# sc-52,658) and β-actin (Cat# sc-47,778) antibodies were purchased from Santa Cruz Biotechnology, Inc. (USA).

### Cell culture and identification

The isolation and identification of primary chondrocytes from SD rats according to the previously described [18, 19]. The experimental protocols were approved by Animal Ethics Committee of Wuhan University (Wuhan, China). SD rats were anesthetized through intraperitoneal injection with 1% sodium pentobarbital (40 mg/kg). After experiment, the animals were then euthanized using overexposure of carbon dioxide (CO_2_). The isolated cartilage was digested by 0.25% trypsin-EDTA (Gibco, USA; Cat# 25200056) and washed twice with PBS (pH 7.4), then 0.2% collagenase type II (Gibco, USA; Cat# 17101015) was added for digestion at 37 °C. Harvested chondrocytes were cultured in complete culture medium containing 10% FBS and 1% penicillin-streptomycin (Gibco, USA; Cat# 15140122). Chondrocytes were identified by immunohistochemistry staining of collagen type-II as previously [20].

### Establishment of apoptotic model and experimental grouping

In this present study, different concentrations (0.5, 1.0, 2.0, 3.0 and 4.0 mM) of sodium nitroprusside (SNP), NO donor, was used to build up the chondrocyte apoptotic model. Our previous results showed the maximum apoptotic response was observed at 3 mM SNP treated chondrocyte [18]. To investigate the protective roles of CMCS on cytotoxicity and apoptosis, chondrocytes pretreated by different doses of SNP followed by addition of CMCS (50, 100 and 200 μg/ml) for further experiments.

### MTT assay

Cell proliferation was determined by MTT (3-(4, 5-dimethylthiazol-2-yl)-2, 5-diphenyltetrazolium bromide) colorimetric analysis according to the manufacturer’s protocol and previously described [21]. Chondrocytes were cultured at the density of 1 × 10^5^ cells in 96 well plates overnight, the treated cells were then incubated with MTT solution at 37 °C for 4 h. The absorbance at 570 nm was recorded by Absorbance Microplate Reader microplate reader (EL × 800, USA). The results were expressed as OD value reduction relative to control group, all assays were conducted in triplicate.

### LDH assay

LDH (lactate dehydrogenase), a soluble cytosolic enzyme, exists in almost all living cells. LDH is expressed extensively in body tissues, it is released during tissue damage, its release into culture medium due to cell plasma membrane damage, the LDH increasing correlated to cell viability. Briefly, chondrocytes were cultured in 96-well plates at density of 1 × 10^5^ cells following by indicated treatment. The absorbance at 490 nm was detected spectrophotometrically using Microplate Reader microplate reader (EL × 800, USA). LDH activity was presented as percentage to control group, experiments were conducted in triplicate.

### Determination of apoptosis by Annexin V-FITC/PI staining

Chondrocyte apoptosis was determined by Annexin V-FITC/PI double labeling according to the manufacturer’s protocol. Briefly, after indicated cultures, chondrocytes were digested and suspended in binding buffer. Then 5 μl Annexin V and 5 μl PI solutions were added into cells and incubated for 15 min. Apoptotic rate was detected by BD FACSVerse™ flow cytometer (Becton Dickinson, Heidelberg, Germany) and analyzed by Cell Quest software (BD Biosciences). Experiments were conducted in triplicate.

### Detection of mitochondrial membrane potential (ΔΨm)

Mitochondrial membrane potential (ΔΨm) of chondrocytes were detected by uptake of Rhodamine 123 (Rho123), the fluorescent and cell-permeant dye, which can interact with negative charges in the inner mitochondrial membrane. The damage to ΔΨm causes the leakage of Rho123 from mitochondria to cytoplasm. Briefly, chondrocytes were treated with SNP or SNP/CMCS for 24 h then treated by Rho123 (10 μg/ml) for 20 min at room temperature, the ΔΨm was observed under excitation/emission (Ex/Em) at 488/510 nm by fluorescence microscope.

### Quantitative real-time polymerase chain reaction (qRT-PCR)

mRNA was isolated from chondrocytes using TRIzol (Invitrogen, USA; Cat# 15596026) and quantified the concentrations by using NanoDrop™ Spectrophotometer (ND-1000; Thermo Fisher Scientific, USA) at 260/280 nm. Reverse transcription was performed using iScript™ Reverse Transcription Supermix (Bio-Rad, USA; Cat# 1708840). PCR reaction was conducted in a volume of 20 μl system in which 2 μl cDNA, 10 μl 2 × SsoAdvanced Universal SYBR Green Supermix (Bio-Rad, USA; Cat# 172–5274), 0.4 μl primer (10 μM) and 7.2 μl ultrapure water was mixed. The reaction was carried out in ABI 7500 Prism Detection System (Applied Biosystems, USA). The data were calculated by using 2^-∆∆CT^ and experiments were conducted in triplicate. The primers are showed in Table 1.

**Table 1:** Primer sequences of target genes

Gene	Primer	Sequence	Product size (bp)
Bcl-2	Forward	TACGAGTGGGATACTGGAGA	165
	Reverse	TCAGGCTGGAAGGAGAAGT	
Bax	Forward	GTTACAGGGTTTCATCCAGG	175
	Reverse	CGTGTCCACGTCAGCAATAC	
Cyt *c*	Forward	AAATGGGTGATGTTGAAGCT	139
	Reverse	TTGGTCCAGTCTTATGCGGCT	
Caspase-3	Forward	CTGGACTGCGGTATTGAGTG	156
	Reverse	GGGTGCGGTAGAGTAAGCG	
GAPDH	Forward	TGTCTCCTGCGACTTCAACAG	256
	Reverse	GAGGCCATGTAGGCCATGAG	

### Western blot analysis

The proteins in chondrocytes were extracted by protein lysis buffer. After samples were quantified and boiled, the equal samples were added into 12% SDS-polyacrylamide gels and separated by electrophoresis, the proteins then transferred to polyvinylidene fluoride (PVDF) membrane (Thermo Scientific, USA; Cat# 88585). Subsequent to blocking with 5% non-fat milk in Tris-buffered saline for 45 min then incubated with primary antibodies overnight. After washing with PBST, the secondary horseradish peroxidase-conjugated antibody (1:2000) was added. The signals were visualized by using electrochemiluminescence on Kodak-X-OMAT-AR film (Kodak, Rochester, NY, USA) with enhanced chemiluminescence (ECL, Pierce, USA; Cat# 32106), the images were captured by Geliance 200 Gel Imaging system. The desnitometry analysis was quantified by using the Imaging J software (Rawak Software, Inc. Germany). The protein expressions were normalized to that of β-actin.

### Statistical analysis

Each experiment was performed at least three times and samples in each group were provided in triplicate. The data and value were presented as the mean ± standard deviation of the mean. Statistical analysis was performed using analysis of variance (ANOVA) by using Statistical Package for Social Sciences (SPSS, version 19.0). The significance of statistical level was set at *P* values less than 0.05.

## Results

### Chondrocytes culture and identification

**Fig. 1:**
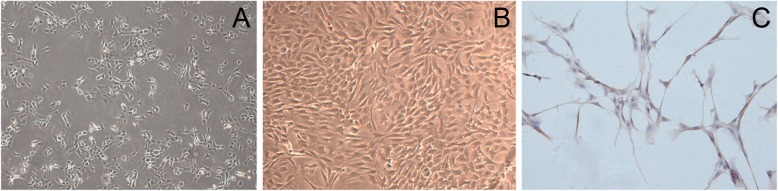
Chondrocytes culture and identification in vitro. Figures of primary cultured chondrocytes for 3 days **a**, 7 days **b** and immunohistochemistry staining by collagen type-II **c**

In our present study, the cultured primary chondrocytes grown as a suspension culture in the beginning of isolation. Cultured overnight, the attached cells can be observed and changed into cycle-like shapes. Cells spread across the culture dish in the long spindle lines exhibited clear boundaries after cultured for 3 days (Fig. 1a). After cultured for 7 days, the cells showed the irregular cobblestone-like shape (Fig. 1b). The cultured cells were identified by immunohistochemistry staining of collagen type-2 (Fig. 1c).

### NO inhibits cell viability in cultured chondrocytes

**Fig. 2:**
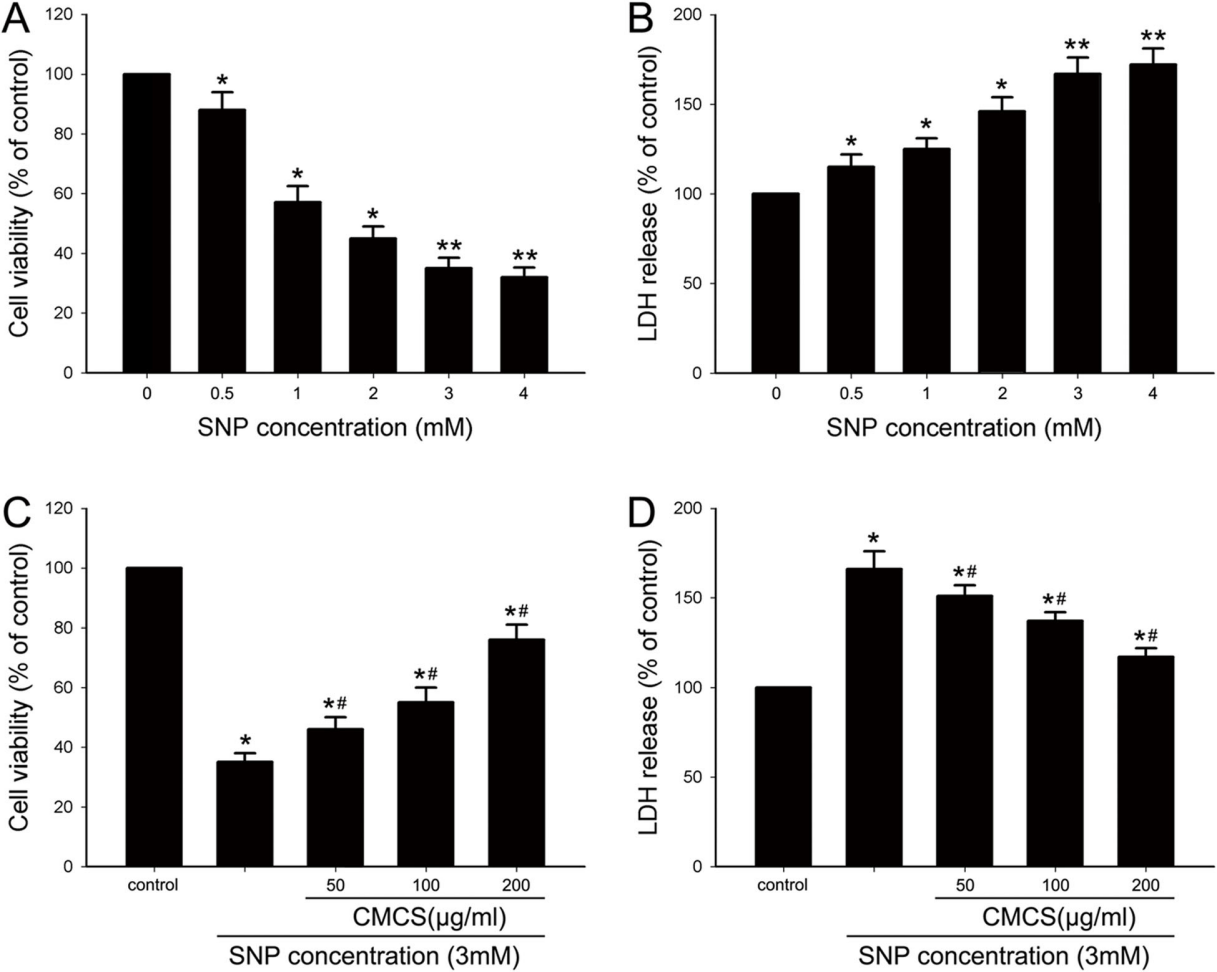
Effects of CMCS on cell proliferation of NO-induced chondrocytes by MTT and LDH assays. **a** Effects of SNP on cell viability in chondrocytes. **b** Effects of SNP on LDH release in chondrocytes. **c** Effects of CMCS on cell proliferation in NO induced chondrocytes. **d** LDH release assay results. *P < 0.05 vs. the control cells, #*P* < 0.05 vs. 3.0 mM SNP treated cells

In this study, SNP (0.5, 1.0, 2.0, 3.0 and 4.0 mM) was used to induce the cultured chondrocytes. The MTT results showed that SNP could inhibit cell viability in cultured chondrocytes with a concentration dependent manner, 3.0~4.0 mM SNP treated cells have the maximum inhibitory response, there was no significant difference between 3.0 and 4.0 mM SNP-treated groups (Fig. 2a). As showed in Fig. 2b, the similar response pattern was observed in LDH release sassy, there was a corresponding gradual increase of LDH release in SNP-induced chondrocytes.

### CMCS increases cell viability in NO-induced chondrocytes

As showed in Fig. 2, the dramatic decrease of cell viability and increase of LDH release were observed in 3.0 mM SNP induced chondrocyte, were 37.43 ± 3.51% and 162.37 ± 10.98%, respectively. The cell viability was increased up to 46.56 ± 4.21%, 56.48 ± 4.91%, 73.42 ± 6.76% (Fig. 2c) and LDH release decreased to 151.22 ± 12.38%, 135.47 ± 10.22%, 116.98 ± 9.72% (Fig. 2d) in 50, 100 and 200 μg/ml CMCS treated group, respectively.

### CMCS protects chondrocytes against NO-induced apoptosis

**Fig. 3:**
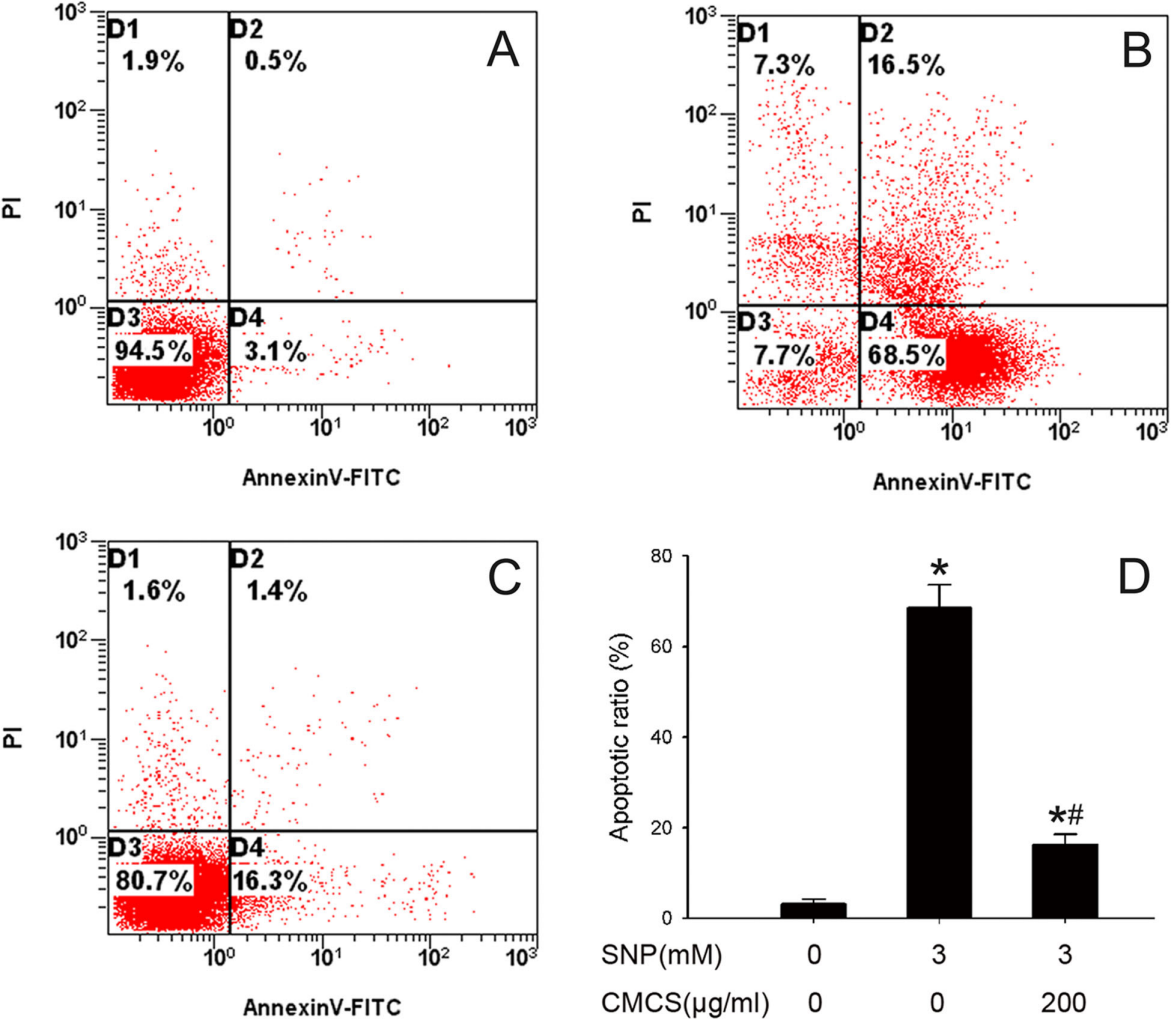
Protective effect of CMCS on NO-induced apoptosis in chondrocytes. Apoptotic cells were detected by FCM. **a** Control group; **b** 3.0 mM SNP treated group; **c** 3.0 mM SNP + 200 μg/ml CMCS treated group; **d** The column results of apoptotic rate. *P < 0.05 vs. the control cells, #*P* < 0.05 vs. 3.0 mM SNP treated cells

Chondrocytes apoptosis was detected by Annexin V-FITC/PI double labelling staining flow cytometry analysis. Apoptotic rate was 68.5% in 3.0 mM SNP-treated chondrocytes (Fig. 3b), control cells were 3.1% (Fig. 3a). The apoptotic rate decreased to 16.3% after treatment with 200 μg/ml CMCS in 3 mM SNP-induced chondrocytes (Fig. 3c). All the results indicated CMCS could protect chondrocyte against NO-induced apoptosis.

### CMCS restores ΔΨm in NO-induced chondrocytes

**Fig. 4:**
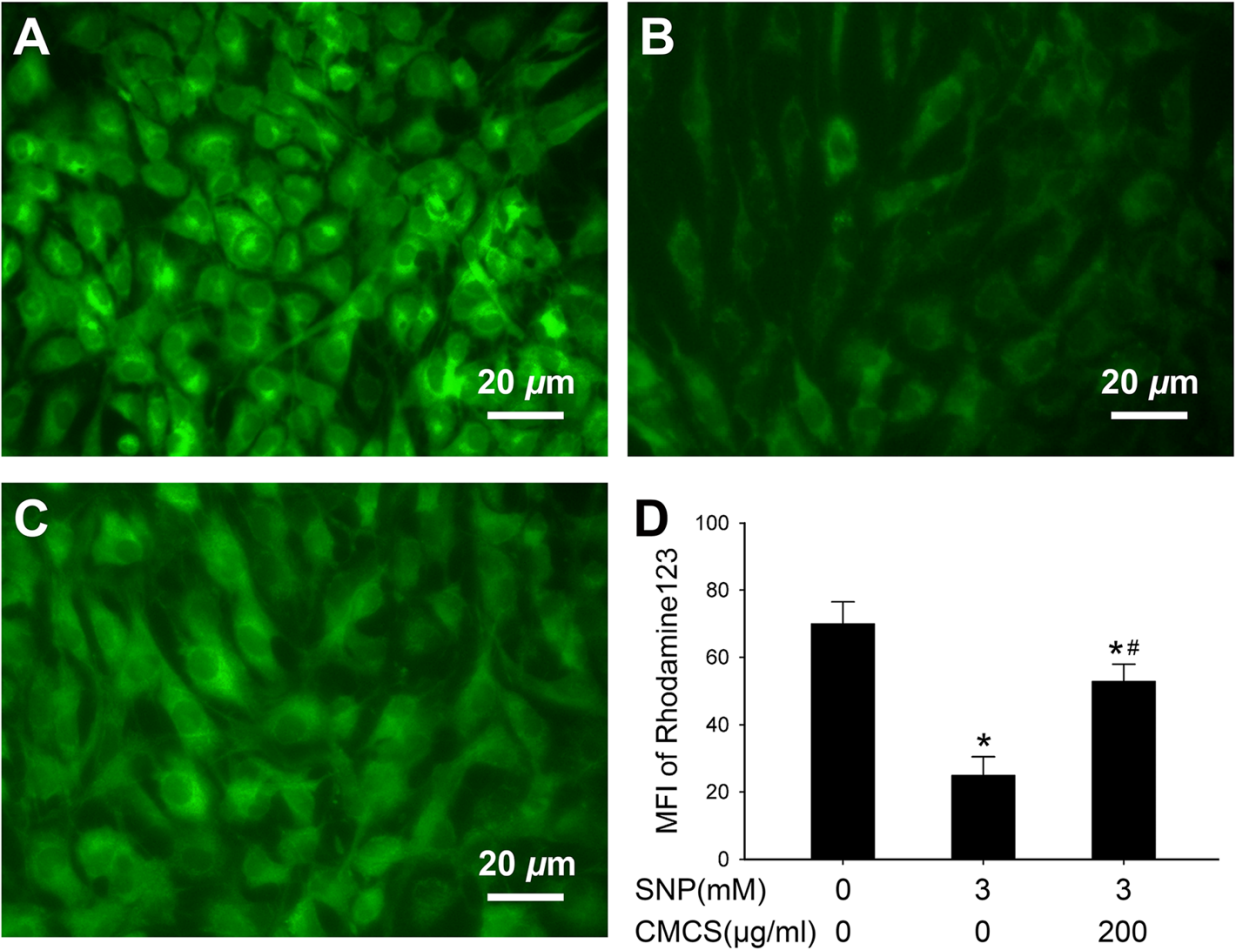
CMCS restores NO-induced ΔΨm in chondrocytes. ΔΨm of chondrocytes was detected by Rho123 staining. **a** Control group; **b** 3.0 mM SNP treated group; **c** 3.0 mM SNP + 200 μg/ml CMCS treated group; **d** The column results of ΔΨm assay. *P < 0.05 vs. the control cells, #*P* < 0.05 vs. 3.0 mM SNP treated cells

In this study, the effects of SNP with or without CMCS on ΔΨm were determined by Rho123 staining. As demonstrated in Fig. 4, 3.0 mM SNP (Fig. 4b) induced a significant reduction of ΔΨm compared with control cells (Fig. 4a), 200 μg/ml CMCS increased the ΔΨm in SNP-induced chondrocytes (Fig. 4c). all above results indicated CMCS has protective effects on mitochondrial function in NO-induced apoptosis of chondrocytes.

### CMCS regulates expression of Bcl-2 and Bax in NO-induced chondrocytes

**Fig. 5:**
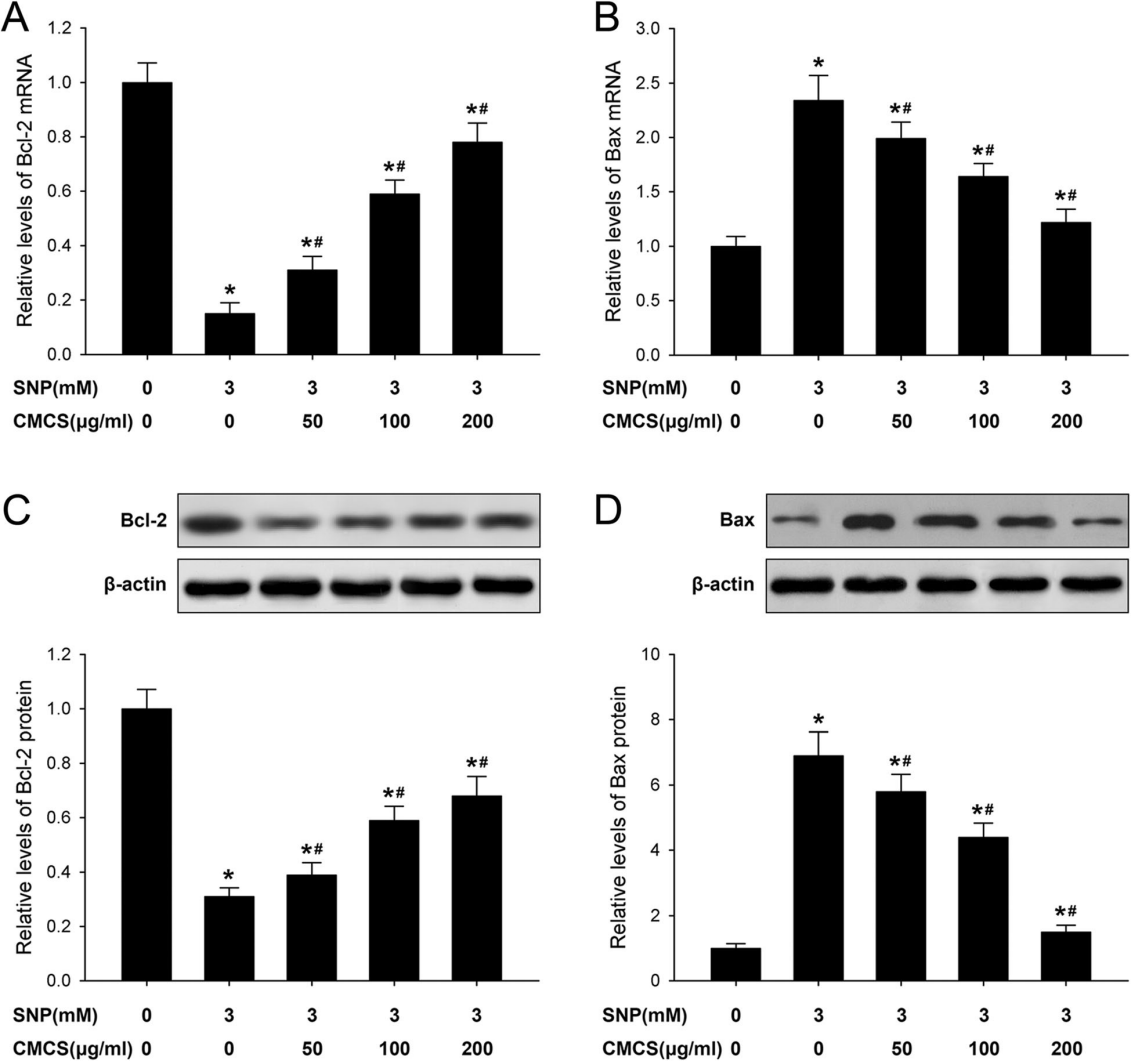
Effect of CMCS on Bcl-2 and Bax expressions in NO induced chondrocyte. **a** Bcl-2 mRNA expressions; **b** Bax mRNA expressions; **c** Bcl-2 protein expressions; **d** Bax protein expressions. Data were normalized to GAPDH or β-action. *P < 0.05 vs. the control cells, #*P* < 0.05 vs. 3.0 mM SNP treated cells

The expression levels of Bcl-2 and Bax were detected by qRT-PCR and western blot, respectively. As illustrated in Fig. 5, the Bcl-2 level was decreased while Bax was increased in 3 mM SNP-induced chondrocytes, these down-regulation and up-regulation expressions were reversed by following treatment with CMCS (50, 100 and 200 μg/ml with dose dependent manner), Bcl-2 was increased (Fig. 5a) and Bax was decreased (Fig. 5b). The western blot results also showed the similar expression patterns of Bcl-2 and Bax, decreased Bcl-2 and increased Bax were observed in 3.0 mM SNP treated group, CMCS (50, 100 and 200 μg/ml) could reverse the expression pattern of Bcl-2 (Fig. 5c) and Bax (Fig. 5d) in SNP-induced chondrocytes. Above results indicated CMCS protect NO-induced apoptosis in chondrocyte via regulating expressions of Bcl-2 and Bax.

### CMCS inhibits Cyt *c* release and caspase-3 activity in NO-induced chondrocytes

**Fig. 6:**
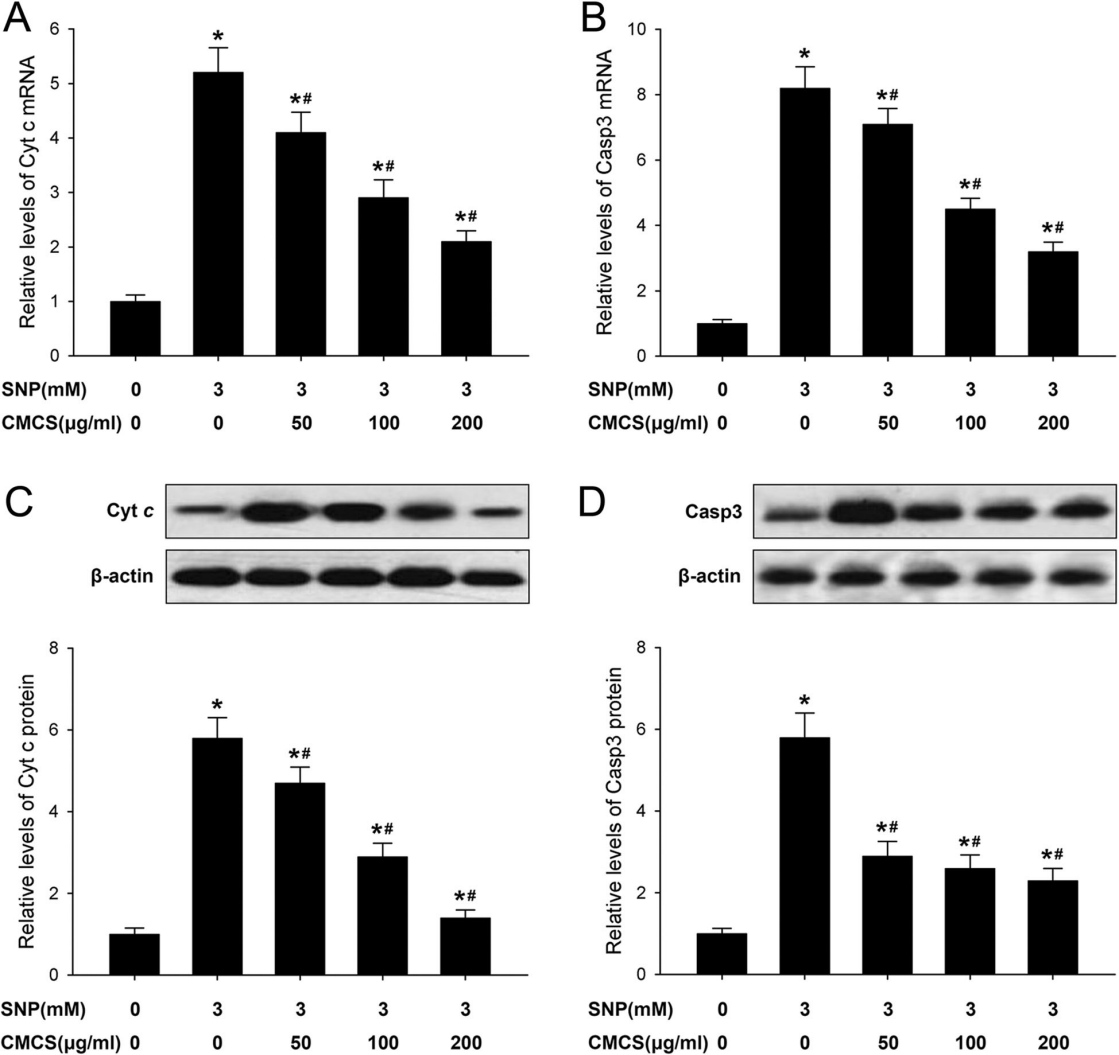
Effects of CMCS on expression of Cyt *c* and activity of caspase-3 activity in NO induced chondrocyte. **a** Cyt c mRNA expressions; **b** caspase-3 mRNA expressions; **c** cytoplasmic Cyt c protein expressions; **d** cleaved caspase-3 protein expressions. Data were noralized to GAPDH or β-action. *P < 0.05 vs. the control cells, #*P* < 0.05 vs. 3.0 mM SNP treated cells

The Cyt *c* release from mitochondria and caspase-3 activation are the critical event during cell apoptosis. In this study, the mRNA and protein expression levels of cytoplasmic Cyt *c* and cleaved caspase-3 were detected by qRT-PCR and western blot analysis, respectively. As showed in Figs. 6, 3.0 mM SNP significantly increased the mRNA expression of Cyt *c* and activity of caspase-3 in cultured chondrocytes, CMCS (50, 100, 200 μg/ml) could down-regulate the expressions of cytoplasmic Cyt *c* (Fig. 6a) and cleaved caspase-3 (Fig. 6b) with the concentration dependent manner. The similar expression patterns of cytoplasmic Cyt *c* and cleaved caspase-3 were also observed in western blot analysis, obvious elevations of Cyt *c* and caspase-3 in 3 mM SNP treated group, after treatment with CMCS (50, 100 and 200 μg/ml), the expression levels of cytoplasmic Cyt *c* (Fig. 6c) and activated caspase-3 (Fig. 6d) were decreased with dose dependent manner. Above results showed the involvement of Cyt *c* release and caspase-3 activation in protective effects of CMCS on NO-induced apoptosis in cultured chondrocytes.

## Discussion

Numerous studies have shown that OA often occurs with the wear and degeneration of the cartilage, and changes in the cartilage are closely related to the occurrence of OA. Chondrocytes are the only cell type in cartilage tissue, play an important role in maintaining the integrity of the structure and function of the articular cartilage. The apoptosis of chondrocytes is closely related to the progression of OA [22, 23]. Thus, inhibition of the chondrocyte apoptosis may be an efficient method for the treatment of OA. The present results showed that CMCS, the soluble derivative of chitosan, inhibited the SNP-induced chondrocyte apoptosis, which indicates that CMCS may be a potential agent for treatment of OA.

OA is a chronic, painful, degenerative and inflammatory disease, and there is no radical therapy available to date. Only several agents, such as non-steroidal anti-inflammatory drugs (NSAIDs) and cyclooxygenase-2 inhibitors, are used for the treatment of OA. However, the long-term use of NSAIDs may be associated with detrimental effects, especially adverse gastrointestinal effects [24]. Thus, novel and more effective therapeutic methods need to be developed for reducing the disease burden of OA.

Nitric oxide (NO) plays an important role in the development and progression of OA via inducing the chondrocytes apoptosis and articular cartilage degeneration, which is a central pathogenic feature of OA, inhibition of NO-induced apoptosis exerts the therapeutic potential in treatment of OA [19, 25, 26]. SNP, as a donor compound for NO, has been known as an ideal agent that induces chondrocytes apoptosis in vitro [27, 28]. In the current study, we established a model of SNP-induced apoptosis of chondrocyte to investigate the anti-apoptotic effect of CMCS. Firstly, treatment with SNP decreased the cell viability of chondrocyte, however, CMCS partly abolished this effect. Secondly, FCM revealed that CMCS effectively prevented chondrocytes apoptosis. Thirdly, impairment of mitochondrial function, such as the loss of mitochondrial membrane potential, is also involved in the process of apoptosis. The results of Rho123 staining verified that CMCS greatly inhibited the SNP-induced collapse of △Ψm. Our results also confirmed that the protective effects of CMCS might be associated with △Ψm recovery and stabilization. Based on these findings, we can deduce that CMCS effectively mitigated the SNP-induced chondrocyte apoptosis through certain intracellular mechanisms.

It is widely recognized that apoptosis or programmed cell death of articular chondrocytes is involved in the pathogenesis of OA, the pivotal role of cell apoptosis in OA has also been confirmed in in vitro and in vivo models [22, 23, 29]. Increased evidence indicates that CMCS could protect chondrocyte against SNP-induced apoptosis [18, 19]. Further, similar results were also obtained from FCM experiment, the apoptotic rate of chondrocytes in the SNP (3 mM) was 68.5%, while co-treatment with CMCS (200 μg/ml) decreased to 16.3% (Fig. 3b and c). All these results indicated that CMCS could inhibit the SNP-induced chondrocyte apoptosis. The loss of △Ψm is an early event in apoptosis and coincides with the activation of caspase. Rho123 is sensitive to △Ψm and can be used to evaluate the △Ψm, the results of this present study indicated that CMCS could alleviate the collapse of △Ψm in SNP-induced chondrocytes (Fig. 4).

The Bcl-2 family proteins, including Bcl-2 and Bax, act as the key regulators and mediators of mitochondrial dependent apoptosis pathway. The Bcl-2 family members can act as anti- or pro- apoptotic regulators, which also may regulate the mitochondrial permeability transition pore or early perturbation of mitochondria. Thus, the balance of Bcl-2 and Bax play the important role in maintaining normal mitochondrial function. Under pathological conditions, the Bcl-2/Bax ratio and △Ψm decreases, thus release the apoptotic inducible factors, such as Cyt *c* and trigger the apoptosis cascade [30]. In this study, the effects of CMCS on the activation of Bcl-2 and Bax were investigated and the results showed that CMCS inhibited the down-regulation of Bcl-2 and the up-regulation of Bax at the mRNA and proteins expression levels in SNP-induced chondrocytes (Fig. 5). Therefore, the balance of the Bcl-2/Bax ratio was partially maintained or restored, thus protecting the mitochondrial function and reducing the occurrence of SNP-induced chondrocyte apoptosis.

The activated caspase cascade play the crucial roles in mitochondrial dependent apoptotic pathway, in the current study, we investigated the effects of CMCS on the activation of caspase-3, which is the major executor of apoptosis cascades [31]. CMCS markedly suppressed the SNP-induced increase in the expression of cleaved caspase-3. Thus, the anti-apoptotic activities of CMCS in chondrocytes were related to the intrinsic mitochondrial dependent apoptosis pathway. Based on these results, we proposed an in vitro model illustrated the regulatory mechanism of CMCS in SNP-induced chondrocyte apoptosis. The probable regulatory mechanisms contributed to the anti-apoptotic effect of CMCS is achieved by inhibiting mitochondrial dependent apoptosis pathway, including changes of △Ψm, as well as the mRNA and protein expression levels of Bcl-2, Bax, Cyt *c* and cleaved caspase-3.

## Conclusions

This present study demonstrated CMCS may suppress SNP-induced chondrocyte apoptosis via mitochondrial dependent pathway, indicating that CMCS could potentially be a novel and effective therapeutic agent for treatment of OA.

## Data Availability

All data generated or analyzed during this study are included in this published article (and its supplementary information files and raw data are available from the corresponding author upon reasonable request).

## Abbreviations

Bax: Bcl-2 associated X protein
Bcl-2: B-cell lymphoma 2
CMCS: Carboxymethylated chitosan
Cyt *c*: cytochrome *c*.
DMEM: Dulbecco’s modified Eagle’s medium
FBS: Fetal bovine serum
FCM: Flow cytometry
LDH: Lactate dehydrogenase
MTT: 3-(4,5-Dimethylthiazol-2-yl)-2,5-diphenyltetrazolium bromide
NO: Nitric oxide
OA: Osteoarthritis
SNP: Sodium nitroprusside
ΔΨm: mitochondrial membrane potential

## References

[1] Glyn-Jones S, Palmer AJ, Agricola R, Price AJ, Vincent TL, Weinans H, Carr AJ (2015). Osteoarthritis Lancet.

[2] Young IC, Chuang ST, Hsu CH, Sun YJ, Liu HC, Chen YS, Lin FH (2017). Protective effects of aucubin on osteoarthritic chondrocyte model induced by hydrogen peroxide and mechanical stimulus. BMC Complement Altern Med.

[3] Prince DE, Greisberg JK (2015). Nitric oxide-associated chondrocyte apoptosis in trauma patients after high-energy lower extremity intra-articular fractures. J Orthop Traumatol.

[4] Xue H, Tu Y, Ma T, Liu X, Wen T, Cai M, Xia Z, Mei J (2015). Lactoferrin inhibits IL-1β-induced chondrocyte apoptosis through AKT1-induced CREB1 activation. Cell Physiol Biochem.

[5] Gu Y, Chen J, Meng Z, Yao J, Ge W, Chen K, Cheng S, Fu J, Peng L, Zhao Y (2017). Diazoxide prevents H2O2-induced chondrocyte apoptosis and cartilage degeneration in a rat model of osteoarthritis by reducing endoplasmic reticulum stress. Biomed Pharmacother.

[6] Ossendorff R, Grad S, Stoddart MJ, Alini M, Schmal H, Südkamp N, Salzmann GM (2018). Autologous chondrocyte implantation in osteoarthritic surroundings: TNFα and its inhibition by Adalimumab in a knee-specific bioreactor. Am J Sports Med.

[7] Qiao Z, Tang J, Wu W, Tang J, Liu M (2019). Acteoside inhibits inflammatory response via JAK/STAT signaling pathway in osteoarthritic rats. BMC Complement Altern Med.

[8] Blanco FJ, Rego-Pérez I (2018). Mitochondria and mitophagy: biosensors for cartilage degradation and osteoarthritis. Osteoarthr Cartil.

[9] Shariatinia Z (2018). Carboxymethyl chitosan: properties and biomedical applications. Int J Biol Macromol.

[10] He B, Liu SQ, Chen Q, Li HH, Ding WJ, Deng M (2011). Carboxymethylated chitosan stimulates proliferation of Schwann cells in vitro via the activation of the ERK and Akt signaling pathways. Eur J Pharmacol.

[11] Tao HY, He B, Liu SQ, Wei AL, Tao FH, Tao HL, Deng WX, Li HH, Chen Q (2013). Effect of carboxymethylated chitosan on the biosynthesis of NGF and activation of the Wnt/β-catenin signaling pathway in the proliferation of Schwann cells. Eur J Pharmacol.

[12] He B, Tao HY, Liu SQ (2014). Neuroprotective effects of carboxymethylated chitosan on hydrogen peroxide induced apoptosis in Schwann cells. Eur J Pharmacol.

[13] He B, Wu F, Fan L, Li XH, Liu Y, Liu YJ, Ding WJ, Deng M, Zhou Y (2018). Carboxymethylated chitosan protects Schwann cells against hydrogen peroxide-induced apoptosis by inhibiting oxidative stress and mitochondria dependent pathway. Eur J Pharmacol.

[14] He B, Tao H, Liu S, Wei A (2015). Protective effect of carboxymethylated chitosan on hydrogen peroxide-induced apoptosis in nucleus pulposus cells. Mol Med Rep.

[15] Lee SY, Wee AS, Lim CK, Abbas AA, Selvaratnam L, Merican AM, Ahmad TS, Kamarul T (2013). Supermacroporous poly (vinyl alcohol)-carboxylmethyl chitosan-poly (ethylene glycol) scaffold: an in vitro and in vivo pre-assessments for cartilage tissue engineering. J Mater Sci Mater Med.

[16] Kong Y, Zhang Y, Zhao X, Wang G, Liu Q (2017). Carboxymethyl-chitosan attenuates inducible nitric oxide synthase and promotes interleukin-10 production in rat chondrocytes. Exp Ther Med.

[17] Li T, Song X, Weng C, Wang X, Gu L, Gong X, Wei Q, Duan X, Yang L, Chen C (2019). Silk fibroin/carboxymethyl chitosan hydrogel with tunable biomechanical properties has application potential as cartilage scaffold. Int J Biol Macromol.

[18] He B, Tao H, Liu S, Wei A, Pan F, Chen R, Li X (2016). Carboxymethylated chitosan protects rat chondrocytes from NO-induced apoptosis via inhibition of the p38/MAPK signaling pathway. Mol Med Rep.

[19] He B, Tao H, Wei A, Liu S, Li X, Chen R (2016). Protection of carboxymethylated chitosan on chondrocytes from nitric oxide-induced apoptosis by regulating phosphatidylinositol 3-kinase/Akt signaling pathway. Biochem Biophys Res Commun.

[20] Xu SY, Li SF, Ni GX (2016). Strenuous treadmill running induces a chondrocyte phenotype in rat Achilles tendons. Med Sci Monit.

[21] Paramee S, Sookkhee S, Sakonwasun C, Na Takuathung M, Mungkornasawakul P, Nimlamool W, Potikanond S (2018). Anti-cancer effects of Kaempferia parviflora on ovarian cancer SKOV3 cells. BMC Complement Altern Med.

[22] Musumeci G, Castrogiovanni P, Trovato FM, Weinberg AM, Al-Wasiyah MK, Alqahtani MH, Mobasheri A (2015). Biomarkers of chondrocyte apoptosis and autophagy in osteoarthritis. Int J Mol Sci.

[23] Hwang HS, Kim HA (2015). Chondrocyte apoptosis in the pathogenesis of osteoarthritis. Int J Mol Sci.

[24] Scarpignato C, Lanas A, Blandizzi C, Lems WF, Hermann M, Hunt RH (2015). International NSAID Consensus Group. Safe prescribing of non-steroidal anti-inflammatory drugs in patients with osteoarthritis--an expert consensus addressing benefits as well as gastrointestinal and cardiovascular risks. BMC Med.

[25] Huh JE, Seo BK, Baek YH, Lee S, Lee JD, Choi DY, Park DS (2012). Standardized butanol fraction of WIN-34B suppresses cartilage destruction via inhibited production of matrix metalloproteinase and inflammatory mediator in osteoarthritis human cartilage explants culture and chondrocytes. BMC Complement Altern Med.

[26] Park JU, Kim SJ, Na CS, Choi CH, Seo CS, Son JK, Kang BY, Kim YR (2016). Chondroprotective and anti-inflammatory effects of ChondroT, a new complex herbal medication. BMC Complement Altern Med.

[27] Zhao P, Cheng J, Geng J, Yang M, Zhang Y, Zhang Q, Wang Y, Lu B (2018). Curcumin protects rabbit articular chondrocytes against sodium nitroprusside-induced apoptosis in vitro. Eur J Pharmacol.

[28] Lin J, Wu G, Chen J, Fu C, Hong X, Li L, Liu X, Wu M (2018). Electroacupuncture inhibits sodium nitroprusside-mediated chondrocyte apoptosis through the mitochondrial pathway. Mol Med Rep.

[29] Charlier E, Relic B, Deroyer C, Malaise O, Neuville S, Collée J, Malaise MG, De Seny D (2016). Insights on molecular mechanisms of chondrocytes death in osteoarthritis. Int J Mol Sci.

[30] Wang Q, Zhang L, Yuan X, Ou Y, Zhu X, Cheng Z, Zhang P, Wu X, Meng Y, Zhang L (2016). The relationship between the Bcl-2/Bax proteins and the mitochondria-mediated apoptosis pathway in the differentiation of adipose-derived stromal cells into neurons. PLoS One.

[31] Asokan SM, Hung TH, Li ZY, Chiang WD, Lin WT (2019). Protein hydrolysate from potato confers hepatic-protection in hamsters against high fat diet induced apoptosis and fibrosis by suppressing Caspase-3 and MMP2/9 and by enhancing Akt-survival pathway. BMC Complement Altern Med.

